# World-Class Innovation, but at What Cost? A Brief Examination of the American Healthcare System

**DOI:** 10.7759/cureus.39922

**Published:** 2023-06-03

**Authors:** Spencer Delfino, Andrew Larson, Daniel Haines, Ryan Grell

**Affiliations:** 1 Anesthesiology and Perioperative Medicine, University of Louisville School of Medicine, Louisville, USA

**Keywords:** health innovation, healthcare quality indicators, rising cost of healthcare, public health policy, global healthcare systems

## Abstract

The American healthcare system, often counted among the world’s best, offers expedient access to a highly subspecialized network of physicians who work at the forefront of developing and utilizing novel, cutting-edge procedures, and medications. Patients typically have access to large numbers of qualified physicians in every metropolitan area and thus are afforded the luxury of individual choice in hospital, physician, and experience. Unfortunately, the costs of maintaining such a system are quite profound, and the higher investments do not pay dividends in health outcomes. Here, we elaborate on the greatest achievement and worst flaw in the American healthcare system.

## Editorial

American healthcare’s greatest achievements

Often regarded as one of the gold standards of healthcare in the world, the American healthcare system boasts an impressive collection of accomplishments. Consisting of a vast network of specialized providers, cutting-edge research, and novel therapies, the American system features personalized treatment plans with an emphasis on patient choice [1]. In almost every scenario, individuals have the autonomy to choose their healthcare providers and the nuances of the care they receive. Moreover, this care is more accessible than ever with the continued expansion of healthcare coverage and the integration of mobile clinics and telehealth. 

The American healthcare system has invested significant resources in medical research and pharmaceutical development which has led to groundbreaking discoveries and improved treatments in a variety of fields [2]. This was well illustrated during the COVID-19 pandemic, where the United States outpaced all other countries in vaccine development and distribution. This $32 million investment has led to millions of lives saved and has created potential avenues for the treatment of other diseases. Additionally, the United States leads the world in new drug and medical device approvals, holds the distinction of having the highest number of Nobel laureates in chemistry and medicine, and produces the second-highest impact of scientific works in the world [3].

The degree of medical specialization in the United States is also among the most advanced in the world. Between the years of 2011 and 2015, nearly 90% of graduating internal medicine residents, the specialty responsible for providing the majority of the medical fellows in the country, were pursuing fellowship subspecialization [4]. This degree of individual academic achievement provides patients with physicians who are highly knowledgeable and well equipped to treat them. The downstream effects of this subspecialization can be demonstrated by improvements in a variety of contexts including in cancer detection and treatment. During their additional training, physicians are exposed to improved early oncologic detection strategies and surgical techniques which has contributed to a 32% reduction in the risk of cancer death compared to 1991. This translates to nearly 3.5 million lives saved [5]. 

Patient-centered care and freedom of choice are major strengths of the United States healthcare system. Patient-centered care focuses on placing patients at the center of healthcare decision-making and considering their values, preferences, and needs [1]. This technique has shown great success in multiple avenues. For instance, a Cochrane review of the patient-centered care model in patients with obstructive sleep apnea showed improved patient adherence as well as significant improvements in patient outcomes [6]. Americans are also awarded the freedom of choice in selecting healthcare providers, specialists, and treatment options. This empowers patients to seek care from providers they trust and have confidence in, and it promotes competition among healthcare providers -- potentially leading to higher quality and more accessible care. Overall, the emphasis on patient-centered care and freedom of choice highlights a commitment to individual patient autonomy and the recognition of patients as active participants in their own healthcare.

The United States is well positioned to be a global leader in the emerging field of personalized medicine. Personalized and precision medicine, utilizing a patient's own DNA to guide treatments and predict potential future diseases, is poised to revolutionize the field of medicine and shape its future [7-8]. By analyzing an individual's genetic makeup, healthcare providers can tailor treatments and interventions to their unique genetic profile, maximizing effectiveness and minimizing adverse effects. This approach holds great promise for improved patient outcomes and enhanced precision in healthcare delivery. America is home to a very large number of experts in this field including Vanderbilt University’s Dr. Dan Roden who is well-known worldwide for his pioneering work on personalized and precision medicine [9]. Through its combination of strong research infrastructure, significant investment in genomic research, and precision medicine initiatives, the United States will continue to innovate at the forefront of this transformative field.

The United States is also implementing new policies to increase accessibility to healthcare insurance and services. The Affordable Care Act (ACA), enacted in 2010, expanded Medicaid, created health insurance marketplaces, and limited insurance denials due to pre-existing conditions. Further supplemented by legislation such as the American Rescue Plan and Inflation Reduction Act, which provide additional subsidies for health insurance coverage, these policies have led to a near year-over-year reduction in the rate of uninsured. From 2013 to 2021 there has been a 38% drop in the number of uninsured patients, and more than four million people have gained coverage in the past year alone [10].

As health insurance accessibility continues to expand, there is an increasingly diverse population of patients to be seen by providers. The United States has implemented an innovative approach to meeting this demand -- telehealth. This modality involves the use of telecommunications technology to provide remote medical services, thus removing the need for physical travel. This is of particular importance to those who do not have adequate means of transportation or access to nearby specialists. The number of Americans utilizing telehealth has grown from 0.3 million in 2010 to 27.6 million in 2022. Furthermore, the number of total telehealth visits has grown from 14 million in 2019 to an estimated 200 million in 2021 [11]. Telehealth has not only helped bridge the gap between patients and providers but also saved patients between $19 and $121 per visit -- primarily by avoiding unnecessary visits to an emergency department [12]. Telehealth is also finding its footing within the hospital walls through means of remote monitoring and clinical decision support, reducing intensive care unit (ICU) mortality from 10.7% to 8.6% [13].

In addition to telehealth, mobile clinics are expanding the reach of medical services. Mobile clinics are customized motor vehicles that are self-contained units capable of traveling to communities to provide medical care. Currently, there are upwards of 2000 mobile clinics providing over 6.5 million clinic visits annually as of 2017 [14]. The most offered services include primary care, prevention screenings, and dental exams. Additionally, specialized services such as mammography, mental health monitoring, and eye exams are being integrated as well. 

Numerous studies have demonstrated the efficacy of mobile clinics with regard to increased rates of screening for infectious illnesses such as human immunodeficiency virus (HIV); increased rates of prenatal care and the subsequent reduction of preterm and low birth weight infants; and improved management of chronic conditions such as hypertension, hypercholesterolemia, and diabetes [14-17].

The United States has ascended to the forefront of medical innovation by investing heavily in research which has led to novel treatments and medical devices. Additionally, it offers a medical education system that fosters highly specialized providers who in turn administer patient-centered care rooted in a patient’s freedom of choice. Finally, access to health insurance and medical services continues to grow as the United States advances policy and implements novel modalities in which to deliver medical care.

American healthcare’s worst flaw

Despite all of the innovation and praise in the American healthcare system, there exists a massive flaw with significant implications -- its cost. The United States of America spent $4.3 trillion, more than $12,500 per person, on healthcare in 2021 [18]. This amount made up more than 18% of the country’s Gross Domestic Product (GDP). The investment disparity between the United States and every other developed nation in the world is quite profound. 

In the 27 member states of the European Union (EU), the political and economic collection of nations totaling more than 500 million citizens, the average nation’s percent GDP expenditure on health care was less than 11% [19]. Germany, the EU’s highest spender in 2021, allocated just 12.8% of its GDP for health care. That percentage averaged to be just $6,700 per person. Two of the EU’s lowest spenders, Ireland and Poland, spent just 6.7% and 6.6% of their overall GDPs. This translated to $5,800 and $2,500 per capita, respectively [18].

America also routinely spends significantly more than its North American neighbors with robust market-based economies. In 2021, for example, Canada spent 11.7% of its GDP, $5,900 per capita, on healthcare-related expenses while Mexico spent 6.2% of its GDP, $1,227 per person [18]. As with most nations in the EU, both Canada and Mexico were able to accomplish this even while offering universal healthcare coverage for their citizens.

Unfortunately, instead of seeing a significant return on this investment, the nation lags significantly behind most of the developed world in a wide range of health outcomes. Among the 38 members of the intergovernmental agency Organization for Economic Co-operation and Development (OECD), the United States is ranked 34th in infant mortality, 32nd in total life expectancy, 32nd in suicide rates, and 38th in total obesity rates [18]. 

Additionally, approximately 8% of Americans are not covered by any form of health insurance [10]. For these patients, as well as the underinsured, the anticipated out-of-pocket costs associated with medical treatment can have a profound effect. A recent poll of a representative sample of Americans with and without insurance found that 41% of respondents reported forgoing a necessary visit to their local emergency department in the past year due to concerns about cost [20]. An additional 26% reported delaying or skipping treatment, and 19% reported postponing the purchase of their medications due to cost. Around 12% of Americans required borrowing money to pay for their medical care.

If these extreme expenditures are not improving health outcomes and are a substantial source of financial burden for citizens, what are they going towards and how can we reduce them? Approximately 30% of all healthcare expenditures in the United States go toward an element that has zero effect on the health of its citizens -- administrative expenses [21]. This figure is five times more than Canada spends, eight times more than the average EU country spends, and nearly 13 times more than Japan spends [18-19]. 

Overall, America spends worth of $3.8 trillion on healthcare expenditures with $1.25 trillion of it allocated to administrative burdens [21]. This amounts to more than two times what the country spends on care for cardiovascular disease, more than three times more than it spends on cancer care, and 100 times more than the Center for Disease Control’s 2024 annual budget [22-23].

Some of the administrative burdens can be tied to the cost of providing cutting-edge therapies, emerging pharmaceuticals, and the amount of choice American healthcare consumers cherish, but some of this cost is widely considered to be wasteful. Numerous nonprofit organizations, governmental officials, and researchers have identified similar opportunities to sustainably lower the cost of healthcare administration by as much as 50% [21]. Consensus opinion suggests that the biggest opportunity for cost savings is through administrative simplification and standardization of areas like pre-authorizations, billing modifiers, claims submissions, credentialing, and medical records [21-22, 24]. By making just a few difficult administrative changes that are inconsequential for patient outcomes, hundreds of billions of dollars could be reallocated to avenues that do affect patient outcomes [21]. These changes could help to transform America’s worst flaw in healthcare delivery to become one of its strengths.
